# Low-Level Laser Therapy in Patients with Complaints of Tinnitus: A Clinical Study

**DOI:** 10.5402/2012/132060

**Published:** 2012-04-09

**Authors:** Ahmed H. Salahaldin, Khalid Abdulhadi, Nihal Najjar, Abdulbari Bener

**Affiliations:** ^1^ENT and Head and Neck Surgery Department, Audiology and Balance Unit, Rumaillah Hospital and Hamad General Hospital, Hamad Medical Corporation, Doha, Qatar; ^2^Department of Medical Statistics & Epidemiology, Hamad General Hospital, Hamad Medical Corporation, Doha, Qatar; ^3^Department of Public Health & Medical Education, Weill Cornell Medical College, New York, NY, USA; ^4^Evidence for Population Health Unit, School of Epidemiology and Health Sciences, The University of Manchester, Manchester, UK

## Abstract

*Objective*. The objective of the study was to investigate the effectiveness of low-level laser therapy (LLLT) in treating patients who were suffering from long-term complaints of tinnitus with well-understood etiology and who were not responding to conventional therapy in Qatar. *Design*. This is a prospective clinical study conducted during the period from May 2010 and February 2011. *Setting*. Audiology Clinic, Outpatient Department, Hamad General Hospital. *Subjects and Methods.* The study included 65 patients aged 15–76 years with chronic unilateral or bilateral tinnitus with a minimum duration of illness of one year. The investigation included 101 ears of 65 patients. A 5 mW laser with a wavelength of 650 nm was applied transmeatally for 20 minutes once daily for 3 months. The study was based on a face-to-face interview with a designed questionnaire that recorded the diagnosis of patients, clinical evaluation and audiometric test results, and side effects of low-level laser therapy (LLLT) and scored their symptoms loudness on five-point scale every two weeks. A decrease of one scale point regarding the loudness duration and degree of annoyance of tinnitus was accepted to represent an improvement; at the same time, a pure tone audiometric test was carried out and the results recorded. In addition, a record of the side effect was taken. *Results.* Over half of the patients (56.9%) had some form of improvement in their tinnitus symptoms. Mild improvement was reported in 33.8% of patients, moderate improvement was reported in 16.9%, and full improvement was reported in 6.15%. Of the patients who reported dizzy spells as a symptom of their tinnitus condition, 27.7% reported mild improvement and 16.9% reported full improvement. Common side effects of LLLT were noted among 20% of patients; however, all of them were mild and disappeared within a few days. *Conclusion.* Low-level laser therapy was found to be useful for treatment of chronic tinnitus.

## 1. Introduction

Low-level laser therapy (LLLT) is a medical treatment that uses low-energy-level lasers or light-emitting diodes to stimulate or inhibit cellular function [1]. US Food and Drug Administration has cleared the use of low-level lasers for the treatment of lymphedema [2] and chronic pain [3, 4]. Clinical applications of LLLT in otolaryngology include management of hyperacusis, phonophobia, diplacusis, and sound distortion [5] and the treatment of tinnitus [6, 7]. LLLT had been found useful in treating chronic impaired hearing, sudden sensorineural hearing impairment [5], and in treating Meniere's disease and some other balance disorders [8].

The exact mechanism is still being explored and debated but it is likely that the mechanism is photochemical rather than heat related [9]. It has been assumed that low-intensity laser irradiation increases cell proliferation [10], synthesis of ATP and collagen [11], and release of growth factor [12, 13]. It also promotes local blood flow in the inner ear and activates repair mechanisms through photochemical and photophysical stimulation of mitochondria in hair cells [14].

LLLT targeting the inner ear had been discussed as a therapeutic procedure for cochlear dysfunction [15], in particular for chronic cochlear tinnitus, but efficacy is still under investigation and previous studies using psychomotor measurements reported a reduction in loudness of tinnitus compared with a placebo [7, 16], while others showed no efficacy for the outcome in double-blind randomized studies [17, 18]. Only one of those studies reported a decrease in tinnitus handicapped inventory (THI) scale after LLLT [8]. Gungor et al. in their study of 45 patients were able to demonstrate decrease of loudness duration and degree of annoyance of tinnitus with effective LLLT in 44.8%, 57.7%, and 55.5% of cases and confirmed the effectiveness of treatment [7].

Meniere's disease (MD) is an inner ear disorder characterized by recurrent episodic vertigo, fluctuating hearing loss, and tinnitus [19, 20]. The incidence is estimated to be between 50 and 350 per 100,000 per year [1]. The natural history of MD is typically variable in intensity and frequency. Initial attacks are often predominantly vestibular, while later attacks are more marked by hearing loss and tinnitus. The disease is usually unilateral. Raised endolymphatic pressure (hydrops) is commonly accepted as the causal condition, although a direct relationship between Meniere's and endolymphatic hydrops remains unproven [8].

The aim of this study was to investigate the effectiveness of LLLT in treating patients suffering from long-term complaint of tinnitus with well-understood etiology and who were not responding to conventional therapy in Qatar.

## 2. Subject and Methods

This is a prospective clinical study that was conducted between May 2010 and February 2011. It included 101 ears of 65 patients aged between 15 and 76 years, with chronic unilateral or bilateral tinnitus with a minimum duration of illness of one year. The patients had Meniere's disease, SSNHL, or SND. Patients were complaining of long-standing tinnitus with minimum duration of one year. The etiology was known, and the patients were not responding to conventional therapy. Seven patients were excluded from the study because of different reasons giving a response rate of 89.2%.

A consecutive sample of patients who complied with the criteria were approached and asked for written informed consent to take part in the study. IRB ethical approval was obtained from the Medical Research Committee of the Hamad Medical Corporation to conduct this study.

Meniere's disease was defined according to the guideline from AAO-HNS committee of hearing and equilibrium 1995 [21]. For SSNHL of ≥30 dB loss at 3 consecutive frequencies occur within 3 days [22] and other patients with other forms of SND were other subjective cases of tinnitus chosen after exclusion of objective causes of tinnitus (somato-sensory causes).

### 2.1. Procedure

LLLT radiation was directed at patients for a period of 20 minutes/day for a period of 3 months. We used Tinnitool (Denmark@ Maur, Switzerland), a diode laser delivering continuous wave laser light with a wavelength of 650 nm. The absolute power output is 5 mW, and the laser energy is transmitted through a laser probe inserted into the external auditory meatus. The laser beam is projected into the tympanic membrane through a 17-degree divergent lens creating a spot size of one square centimeter. Time of irradiation was 20 minutes/day resulting in an energy density of about 6 J at the tympanic membrane.

Prior to the application of LLLT, baseline data were collected. First a questionnaire form regarding the general complaint was filled out and a subjective evaluation of the suffering was recorded. Then the following clinical examinations were conducted: ENT, otoneurology, dental, TM joint evaluation, thyroid gland check, auscultation of carotid and vertebral vessels and tributaries. Thereafter, audiological evaluation (audiometry), tympanometry, otoacoustic emission testing, VCNG and caloric testing, and the auditory braistem evoked response study were conducted. Thereafter, laboratory and radiological testing as well as Doppler study, MRI, and Angio testing was conducted. Finally, the patients were asked to fill out a tinnitus handicap inventory questionnaire.

### 2.2. Follow-Up and Outcome Measurement

Two weekly follow-ups and check-ups were conducted by the author in the audiology clinic including clinical evaluation and audiometric testing using (AP61-GSI type A, USA made) audiometer. (AZ26-GSI USA made) tympanometer. As well as (Charter-CSI make ABR system) located in the outpatient setting of Hamad Medical Hospital and (Hortmann VCNG and Caloric system) for the diagnosis of vestibular disorders located in Hamad Medical City Audiology unit. A structured questionnaire was also filled on each follow-up.

Improvement in tinnitus was considered in two ways. Subjective improvement was noted and tabulated in percentage improvement following grading suggested by Prochazka [23] with some modification by the author from the tinnitus handicap infirmary as follows: grade 1: no tinnitus; grade 2: no interfering sound perceived during the day, except in evening; grade 3: interfering sound perceived during the day, interrupting drowse only; grade 4: interrupting drowse and sleep, interfering sound causing discomfort; grade 5: unbearable discomfort, interfering with all activities. 

The data were coded and entered into a computer and processed using the Statistical Packages for Social Sciences (SPSS). Student's *t*-test was used to ascertain the significance of differences between mean values of two continuous variables, and Mann-Whitney test was used for nonparametric distribution. Chi-square analysis was performed to test for differences in proportions of categorical variables between two or more groups. In 2 × 2 tables, Fisher's exact test (two-tailed) replaced the chi-square test if the assumptions underlying chi-square were violated, namely, in case of small sample size and where the expected frequency is less than 5 in any of the cells. Pearson's correlation coefficient was used to evaluate the strength association between two continuous variables. *P* < 0.05 was considered as the cut-off value for significance. 

## 3. Results

Table 1 shows the frequency distribution of the patients according to the cause of tinnitus. A total of 65 patients participated in the study with 29.2% with Meniere's Disease, 23.1% with sudden sensorineural hearing loss, and 49.2% with tinnitus associated with sensorineural hearing loss of other cause.

Table 2 presents the 3-month audiometric response with LLLT treatment of all 65 patients (101 ears). It revealed an improvement of hearing of around 8 dB for low and high frequencies of 44 and 39 audiograms and 5 dB in 41 audiograms. The table also shows no response to 21, 23, and 21 audiograms with 3 dB deterioration of hearing in 20, 18, and 27 audiograms. 

Table 3 shows the results of subjective improvement. 37 patients (56.9%) showed some form of improvement: mild (33.8%), moderate (16.9%), and full improvement (6.2%), while 28 patients (43.1%) had no improvement in their tinnitus condition.

Table 4 displays the side effects encountered during the use of Tinnitool LLLT unit in our study patients. They comprised the common side effects such as itching, red spots, congestion in the deep external auditory canal wall, and mild allergic manifestation. Increased tinnitus and hyperacusis tend to be reported before, but as a whole almost all symptoms were mild and did not warrant treatment as they disappeared in few days.

Table 5 presents the percentage improvement in dizzy spells among our series. Most subjects witnessed some sort of improvement. Full improvement was noted in 11 patients (16.92%). No improvement was noticed in 15 patients (23.07%) and deterioration of dizziness was only noticed in 2 patients (3.07%). 17 patients (26.16%) had no dizziness to begin with at the start of the study.

## 4. Discussion

This prospective clinical study of the effect of LLLT on one of the rather difficult problems to treat “tinnitus” sounds very rewarding. The study showed significant improvement of hearing threshold level in patients with tinnitus, namely, in cases of Meniere's disease and SSNHL and in other patients with tinnitus due to sensorineural hearing loss (49.2%). This was effective in improving hearing by a variable level ranging from 3 dB to 90 dB. We witnessed improvement with reduction in loudness of tinnitus and annoyance in 37 patients amounting to complete disappearance of tinnitus in 4 patients.

In this study, three well-known causes of tinnitus and dizziness in Qatar are chosen including Meniere's disease (MD), sudden sensorineural hearing loss (SSNHL), and tinnitus associated with sensorineural hearing impairment (SND) of different or other causes. All of these conditions can end in prolonged and persistent tinnitus of subjective nature, which may result in annoyance that may interfere with patient's general well-being and social life.

At the same time we observed good improvement in the episodes of dizziness in both patients with MD and SSNHL. The result is more remarkable in patients who complained of dizziness to begin with. It showed reduction in dizziness complaint in around half of the patients under study. Most of these side effects were already described by Prochazka [23].

On the other hand, sudden sensorineural hearing loss (SSNHL) is another clinical entity that we came across in which the etiology is rather obscure and a number of hospital regimes had been advocated as treatment, probably the most successful is combined oral treatment with steroid; in Hamad General Hospital the regimen includes oral steroid, antiviral, and Betahistine. The results range from full improvement (full recovery) in around 37.6% of patients to moderate improvement in 9.5% of patients, to mild improvement in 9.1% of cases [22]. Tinnitus associated with sensorineural hearing loss of cochlear pathology was the 3rd category of tinnitus used in the study.

Our results correspond with results published in the literature on LLLT. For instance, Gungor et al. [7] studied 45 patients (66 ears) with LLLT and demonstrated an improvement grading of 48.8%, 57.5%, and 55.5% of loudness, duration and degree of tinnitus. We also had similar results to those of Wilden [15] with 67% improvement and to those of Marti's [24] study of 18 patients with full improvement in 61%, moderate improvement in 33%, and minor improvement in 6%. Mioc and Mycek [25] selected patients for laser-based therapies in otolaryngology and with high performance improvement. Similarly, Juberg [26] in Norway demonstrated that 87% of their patients reported noticeable improvement and 60% reduction of more than 40% of symptoms as well as no change in 13% of patients.

## 5. Conclusion and Recommendation

LLLT was effective in producing a reasonable improvement in patients' complaints of long-standing tinnitus despite previous treatment of the condition. In addition, it was useful in reducing dizziness that patients may have as a result of their illness. Considering that the side effects are very mild and that over half of the patients had some improvement in their symptoms, it is clear that LLLT is a useful treatment for chronic tinnitus patients. Thus, it is highly recommended that this treatment be introduced to Qatar in order to treat the difficult symptoms of tinnitus patients.

## Figures and Tables

**Table 1:** Frequency distribution of the diagnosis of patients under study *n* = 65 (101 ears).

Tinnitus cause	Number of patients
Meniere's disease	19
SSNHL	15
Associated with SND	32
Tinnitus due to SND with causes other than above*	6

Total number	65

*Included patients with tinnitus due to noise trauma, patients with tinnitus due to cochlear otosclerosis, a patient with postmastoidectomy SND, a patient with bost skull base fracture, a patient with acoustic neuroma, and a patient with a normal audiogram.

**Table 2:** Improvement of hearing confirmed on audiometric improvement at the end of the 3-month study.

Response	Grade	Level	dB change
Improved	Low freq.	44	5, 3, 7, 35, 10, 10, 25, 8, 13, 10, 5, 7.15, 32, 2, 23, 2, 15, 5, 15, 2, 2, 3, 3, 12, 3, 3, 3, 2,10, 15, 3, 2, 23, 23, 2, 5, 27, 2, 80, 15, 7
	Mid freq.	43	2, 5, 20, 2, 8, 2, 2, 5, 5, 13, 2, 2, 25, 50, 5, 3, 18, 3, 18, 2, 3, 7, 12, 10, 3, 5, 3, 10, 2, 2, 5.50.2.23, 5, 5, 3, 90, 5, 5, 33.
	High freq.	30	5, 8, 8, 5, 10.2, 8, 5, 30, 8, 10.2, 27, 72, 2, 7, 43, 5, 8, 8, 3, 6, 2, 5, 5, 30, 17, 3, 2, 63, 25, 8, 13, 13, 8, 18, 90, 5, 90
No change	Low freq.	21	0
	Mid freq.	23	0
	High freq.	21	0

Deteriorated	Low freq.	20	1, 2, 7, 2, 3, 3, 2, 3, 10, 2, 5, 3, 50, 3, 3, 3, 8, 18, 5, 5, 5, 5
	Mid freq.	18	2, 23, 5, 2, 10, 3, 2, 43, 2, 3, 10, 2, 2, 8, 5
	High freq.	27	33, 10, 5, 3, 8, 3, 5, 2, 3, 5, 7, 40, 5, 10, 10, 7, 28, 5.2, 10, 3, 3, 15, 15, 8, 5

**Table 3:** Subjective interpretation of results (*n* = 65).

Subjective improvement	%	Grade	Patients *n* (%)
No improvement	0%	0	28 (43.1%)
Mild Improvement	20–50	1	22 (33.8%)
Moderate Improvement	50–75	2	11 (16.9%)
Full Improvement	75–100	3	4 (6.15%)

**Table 4:** Side effects of LLLT among the studied subjects.

Side effect	AGE (years)	Gender
Itching and red spot in both ears	51	Male
Mild headache	50	Male
Congestion posteroinferior EAM	39	Male
Hearing of sound with metallic resonance and heat in treated ear	59	Male
Earache on exposure to loud sound	47	Male
Conjunctival swelling in the right eye	58	Male
Numbness in forehead and ears	47	Male
Urticarial rash on the external surface of the right arm	56	Female
Papillomacular skin rash (body)	37	Male
Allergic skin rash on the back of the body and hematuria	53	Male
Excessive heat in the right ear	76	Male
Increase in the tinnitus	36	Male
Increase in hearing loss	55	Male

**Table 5:** Improvement in dizzy spells among patients on LLLT (*n* = 65).

Grade	Improvement	Patients number *n* (%)
Grade 0	No improvement	15 (23.07)
Grade 1	Mild improvement	18 (27.69)
Grade 2	Moderate improvement	2 (3.07)
Grade 3	Full improvement	11 (16.92)
Grade 4	Deteriorated	2 (3.07)
Grade 5	Having no dizziness to begin with	17 (26.16)
